# Exploiting DNA Replication Stress as a Therapeutic Strategy for Breast Cancer

**DOI:** 10.3390/biomedicines10112775

**Published:** 2022-11-01

**Authors:** Jing Zhang, Doug W. Chan, Shiaw-Yih Lin

**Affiliations:** Department of Systems Biology, The University of Texas MD Anderson Cancer Center, Houston, TX 77030, USA

**Keywords:** breast cancer, replication stress, replication stress response, DNA replication, chemotherapy

## Abstract

Proliferating cells rely on DNA replication to ensure accurate genome duplication. Cancer cells, including breast cancer cells, exhibit elevated replication stress (RS) due to the uncontrolled oncogenic activation, loss of key tumor suppressors, and defects in the DNA repair machinery. This intrinsic vulnerability provides a great opportunity for therapeutic exploitation. An increasing number of drug candidates targeting RS in breast cancer are demonstrating promising efficacy in preclinical and early clinical trials. However, unresolved challenges lie in balancing the toxicity of these drugs while maintaining clinical efficacy. Furthermore, biomarkers of RS are urgently required to guide patient selection. In this review, we introduce the concept of targeting RS, detail the current therapies that target RS, and highlight the integration of RS with immunotherapies for breast cancer treatment. Additionally, we discuss the potential biomarkers to optimizing the efficacy of these therapies. Together, the continuous advances in our knowledge of targeting RS would benefit more patients with breast cancer.

## 1. Introduction

DNA replication in eukaryotic cells is a multifaceted process, which depends on the activation of numerous signaling pathways to accurately replicate the genome [1]. This process is constantly challenged by events of endogenous or exogenous origin that impede the rate and fidelity of DNA synthesis, thus affecting the integrity of genome. These events, collectively termed replication stress (RS), include DNA lesions such as DNA single-stranded or double-stranded breaks, unusual DNA secondary structures, RNA–DNA hybrids, deficiencies in nucleotide levels, oncogene activation, chromatin inaccessibility and limitation of essential replication factors [2]. In response to RS, cells elicit the DNA damage response (DDR) and subsequently inhibition of cell-cycle progression, which is known as the replication stress response (RSR). The purpose of RSR is to slow DNA synthesis and replication to allow time for DNA repair [3]. The RSR is primarily coordinated by two signaling cascades: ataxia telangiectasia and Rad3-related (ATR)–checkpoint kinase 1 (CHK1) pathway and ataxia telangiectasia mutated (ATM)–checkpoint kinase 2 (CHK2) pathway [4,5,6,7]. Cells rely on these coordinated pathways to prevent mitosis in the presence of DNA damage [8]. Defects in RSR allow for high levels of DNA damage and genomic instability that not only alter gene function, but also lead to continuous proliferation, ultimately provoking carcinogenesis and tumor progression [9,10,11,12,13].

Breast cancer is one of the most frequently diagnosed neoplasms worldwide, with one-third of these patients subsequently dying of this disease. In this heterogeneous disease, an important cause of replication stress is overexpression or constitutive activation of oncogenes, loss of key tumor suppressors, and defects in the DNA repair machinery [2]. Over the past decades, treatments for breast cancer have advanced considerably with effective therapies targeting the estrogen receptor (ER), progesterone receptor (PR), and human epidermal growth factor receptor-2 (HER2). However, patients with triple-negative breast cancer (TNBC), i.e., estrogen receptor-negative, progesterone receptor-negative and HER2-negative, fail to benefit from these treatments. Notably, it was reported that TNBC has high levels of RS due to the activation of various oncogenes and germline BRCA mutations or “BRCAness” in the absence of BRCA mutations [14]. BRCAness is defined as a defect in double-strand break repair (DSBR) via homologous recombination repair (HRR) [15,16]. In the HRR process, recombinase Rad51 interacts with BRCA1 and BRCA2 to perform the search for homologous DNA sequences [16,17]. It has been shown that most hereditary breast cancers are associated with abnormal BRCA1 and BRCA2 genes [18]. Recently, Baldeyron et al. showed that TIPIN depletion results in apoptosis in breast cancer cells by enhancing replication stress [19]. In this context, breast cancer cells evolve various replication stress-resolving mechanisms to avoid toxic levels of replication stress for their survival. Thus, understanding the mechanisms that breast cancer cells have developed to deal with RS could provide promising therapeutic opportunities.

Due to the dependency of cancer cells on the replication stress response for survival, small molecular agents targeting DNA replication stress are under development and have shown antitumor activity in preclinical and clinical studies [20,21]. Cancer cells can be pushed toward apoptosis by introducing further DNA damage by targeting key kinases in RSR, or by using RS to stimulate innate immune response. In this review, we summarize recently gained insights into the mechanisms of the cellular response to RS, discuss the promising therapeutic strategies centered on targeting DNA replication stress in breast cancer, and highlight the potential biomarkers to optimizing the efficacy of these therapies.

## 2. Mechanisms of the Cellular Response to Replication Stress and Rationale in Breast Cancer Therapy

**Figure 1:**
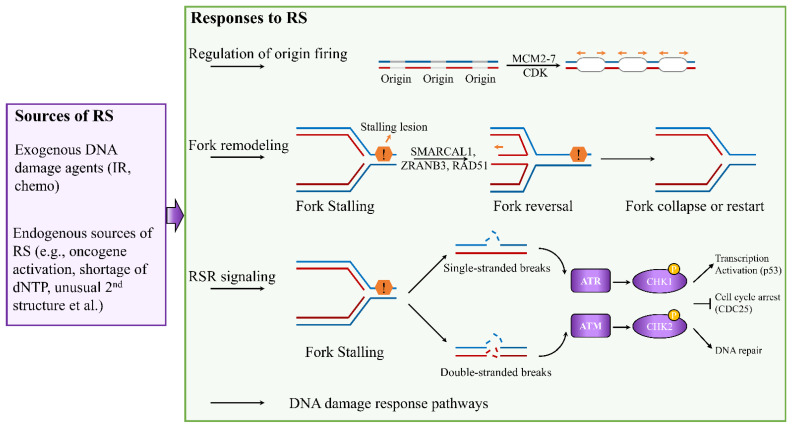
Cellular responses to DNA replication stress. Depending on the types of RS, cells activate numerous responses to ensure accurate completion of genome duplication, including regulation of origin firing, replication fork remodeling, activation of RSR signaling, and involvement of DNA damage response pathways.

Accurate DNA replication is critical to ensure genomic integrity. The fidelity of this process is often challenged by RS, leading to altered replication fork progression and generation of DNA breaks [2,22]. Depending on the types of RS, cells activate four major responses to ensure accurate genome duplication: (1) regulation of origin firing, (2) remodeling of replication forks, (3) activation of replication stress response signaling, and (4) deployment of DNA damage response pathways (Figure 1) [23,24,25].

Eukaryotic genomes are duplicated exactly once during the S phases of each cell cycle. To control replication initiation, the replicative DNA helicase minichromosome maintenance complex 2–7 (MCM2–7) is loaded at replication origins during G1 phase, and activated only during the S phase [6]. This process is called licensing. Activation of licensed origins, which is known as origin firing, requires the activities of cyclin-dependent kinase (CDK) [6]. Deregulation of origin activation can generate replication stress in breast cancer. For example, high levels of MCM2 are associated with poor survival in patients with breast cancer [26,27]. Furthermore, Issac et al. have showed that increased protein expression of MCM2, MCM4, and MCM6 is associated with luminal B, HER2-positive, and TNBC [28]. These MCM proteins may serve as potential treatment targets for breast cancer patients. Unscheduled replication is another source of replication stress in breast cancer. This occurs when the timing of origin activation is altered, leading to DNA regions replicating more than once in one cell cycle or an increase in origin firing in early S phase [6]. Unscheduled replication occurs under the upregulation of DNA replication factor CDT1 and cell division control protein 6 (CDC6). High levels of CDT1 and CDC6 are associated with poorer survival in the breast cancer patients, suggesting that CDT1 and CDC6 are potential therapeutic targets for treatment of breast cancer [29]. To date, the mechanism that regulate origin firing remain largely unknown, and few drugs targeting these key proteins are in the clinical trials. Thus, better understanding the regulation of the origin firing can provide novel therapeutic strategies in the treatment of breast cancer.

Replication fork remodeling is a common response to RS, which involves unwinding of newly synthesized strands and annealing of parental strands. In this remodeling process, stalled forks are converted into four-way junctions to facilitate DNA damage repair [24]. Many key factors involved in reversed fork restart have been identified, including ZRANB3 and SMARCAL1 in reversed fork formation, and BRCA1 and BRCA2 in reversed fork protection [30,31]. RAD51, well-known for catalyzing strand invasion in homologous recombination (HR) repair of DNA double-strand breaks, also plays an important role in regulating replication fork reversal [23]. Furthermore, PARP1 has been linked to recruitment of MRE11 to stalled forks and regulation of fork restart to restore replication fork stability [32,33]. Similar to PARP1, RAD52 can promote the recruitment of MRE11 to stalled replication forks and fork degradation [34,35]. Additionally, DNA2, which functions in dsDNA break repair, has been shown to assist Werner syndrome helicase WRN in controlling HR-mediated restart of reversed replication forks [36,37]. Recently, Dibitetto et al. identified the essential role of DNA-dependent protein kinase catalytic subunit (DNA-PKcs) in promoting fork reversal and preventing genome instability [38]. They found that DNA-PKcs inhibition in BRCA2-deficient breast cancer with acquired PARPi resistance efficiently restored drug sensitivity by impairing fork slowing. The identification of these key proteins in replication fork remodeling provides great opportunities to target RS in cancer therapy.

To cope with RS, cells can also rely on RSR cascade to arrest the cell cycle, protect stalled forks, and allow time to repair the replication fork (Figure 1). RS can perturb the coupling between the replicative helicase and polymerases, resulting in the uncoupling of leading- and lagging-strand synthesis, which generates double-strand breaks (DSBs) and/or single-strand DNA (ssDNA) gaps [6]. DSBs primarily trigger activation of ATM and DNA-dependent protein kinase (DNA-PK), whereas ssDNA coated with replication protein A (RPA) activates ATR via ATR-interacting protein (ATRIP) [39,40,41]. ATR and ATM are apical checkpoint kinases, which regulate the cellular response to replication fork blockage and DNA damage [42]. These kinases activate the effector checkpoint kinases CHK1 and CHK2, respectively, and they regulate the timing of replication origin firing independently of DNA damage [42]. Activation of the ATR–CHK1 pathway leads to cell-cycle arrest by inactivating the cell division cycle 25 (CDC25) phosphatase family or through WEE1 kinase [43,44]. This ATR–CHK1 signaling also promotes replication fork stabilization and restart [45]. Furthermore, H2AX, phosphorylated in an ATR-dependent manner in response to replication stress, recruits RAD51 at reversed forks to protect synthesized DNA degradation and assists with fork restart [46]. Both CHK1 and CHK2 can regulate transcription activation drive by p53 [6]. In addition to replication fork remodeling and RSR signaling, several DDR pathways are activated at stalled forks to allow for recovery from the RS [47,48]. Together, these steps are essential to ensure timely completion of DNA replication and maintain genome stability.

**Figure 2:**
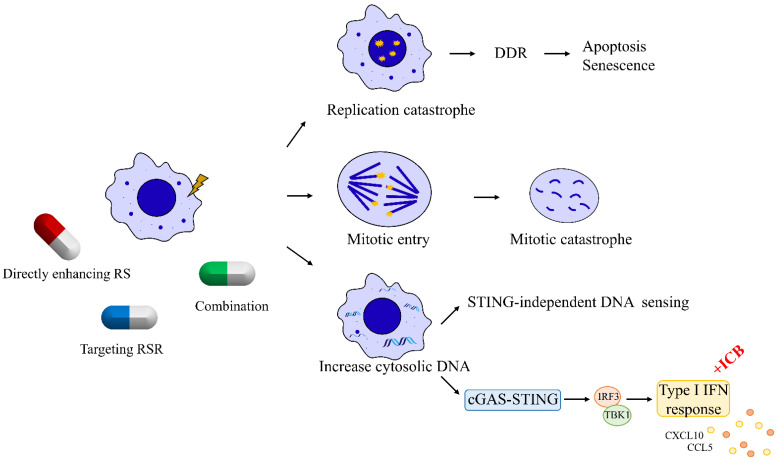
Targeting DNA replication stress in breast cancer. Breast cancer cells harboring RS can be targeted through three major mechanisms. First, RS can be harnessed in breast cancer cells with high intrinsic RS to induce replication catastrophe. Second, breast cancer cells can be targeted by abrogating their G2–M cell-cycle checkpoint to induce mitotic catastrophe. Third, breast cancer cells with high intrinsic RS exhibit high levels of cytosolic DNA leaking from nucleus, which can trigger interferon signaling to prime tumor for immune therapies.

Failure to remove replication stressors due to the loss of replication stress response and repair proteins is a prominent feature of tumor cells. Because of this key feature that distinguishes cancer cells from normal cells, cancer cells harboring replication stress can be targeted through three major mechanisms (Figure 2). First, cancer cells can be pushed toward cell death by enhancing replication stress to induce replication catastrophe [21,49]. Second, cancer cells can be targeted by inhibiting the key kinases of the RSR cascade that cells depend upon to survive, such as ATR, ATM, CHK1, WEE1, and DNA-PK. Inhibition of these key mediators ultimately promotes the premature entry of tumor cells into mitosis, inducing mitotic catastrophe [7,49]. Third, replication stress due to chemotherapies or impaired DNA repair factors induces DNA release from the nucleus to cytoplasm. These DNA fragments trigger cytosolic DNA sensing and interferon signaling, which subsequently prime tumor for immune therapies [7,50,51,52]. The formation of micronuclei has been shown to be a key source of immunostimulatory DNA in cells lacking RNase H2 [53]. However, the release of ssDNA fragment directly from stalled replication forks in TREX1- or SAMHD1-deficient cells has also been shown to induce interferon signaling [51,52]. Several aspects of these mechanisms such as the interplay of proteins in response to RS to promote DNA fragments release from the nucleus into the cytoplasm have yet to be defined.

## 3. Therapeutic Strategies That Induce Replication Catastrophe in Breast Cancer

Since the persistence of replication stress is observed almost exclusively in cancer cells, enhancing replication stress can paradoxically lead to cell death by introducing further DNA damage in a catastrophic manner. Many traditional chemotherapeutic agents, acting by increasing the endogenous replication stress within breast cancer cells, have been developed and have shown antitumor activity (Table 1). For instance, gemcitabine, a deoxycytidine analogue, leads to a delay in replication fork progression by inhibiting ribonucleotide reductase and by competing with dCTP for incorporation into newly synthesized DNA [54]. Another nucleoside analogue 5-fluorouracil, frequently used to treat breast cancer, functions mainly by inhibition of thymidylate synthetase to reduce the amount of thymidine for DNA replication [55,56]. TAS1553, a small-molecule subunit interaction inhibitor of ribonucleotide reductase, has shown antiproliferative activity in breast cancer cells by dramatically reducing the intracellular dATP pool and causing DNA replication stress [57].

Unlike nucleoside analogues, which reduce dNTP pools to direct inhibition of DNA synthesis, alkylating agents and platinum-containing compounds increase the replication stress in breast cancer cells by directly modifying DNA through attacking the DNA bases forming covalent DNA adducts [83,84]. The formation of DNA adducts interferes with the progression of DNA polymerases, which results in delay replication fork progression. In addition to these RS inducers, topoisomerase inhibitors can increase replication stress by promoting R-loop formation, leading to global replication fork slowdown [85]. Another class of replication stress inducing agents that target breast cancer cells by directly inducing DNA damage is represented by poly(ADP-ribose) polymerase (PARP) inhibitors. PARP inhibitors are believed to interfere with the replication machinery and promote fork collapse by trapping PARP on DNA [86,87]. Additionally, PARP inhibitors cause an accumulation of single-strand breaks (SSBs) or Okazaki fragment processing [88,89]. More recently, PARP inhibitors were reported to increase the speed of fork elongation and amplify the replication stress in breast cancer cells [90].

Given that DNA polymerases play key roles in replication stress sensing upstream of ATR, inhibitors targeting DNA polymerases are a novel and attractive class of drugs that are in preclinical development. CD437, targeting POLA1 which encodes DNA polymerase α, has been shown to induce apoptosis in breast cancer cells but not in normal cells [67,91]. Additionally, a small-molecule inhibitor of DNA polymerase δ, zelpolib, displays superior antiproliferative properties in triple-negative breast cancer cells by inhibiting DNA replication and enhances the sensitivity of HR-proficient cells to PARP inhibitors [68]. Although these preclinical studies demonstrate the potential of these agents, further clinical evaluation is required to determine their efficiency.

In addition, understanding the collaboration among proteins that regulate replication fork remodeling provides novel therapeutic targets for capitalizing on endogenous replication stress in breast cancer (Table 1). DNA2 has been shown to play important roles in processing and restarting of reversed replication forks [36]. A selective small-molecule DNA2 inhibitor C5 has been reported to inhibit resection at stalled forks, as well as reduce recombination [69]. This compound is more potent in breast cancer cells defective in replication fork protection and sensitizes cells to traditional RS-inducing drugs. Recently, another DNA2 inhibitor NSC-105808 was identified to sensitize breast cancer cells to oncogene-induced replication stress [37]. However, these studies are still in their early stages. Whether these proteins involved in replication fork remodeling might be successfully developed as therapeutic targets, and what the therapeutic efficiency of these new drugs might remain to be investigated.

While these replication stress inducers target breast cancer directly, their efficacy is limited due to their associated toxicities and the rapid emergence of resistance. Thus, synergistic effects of two different replication stress inducers have been investigated. For example, combinations of PARPi and other replication stress inducers, such as cisplatin [92,93], carboplatin [94,95], temozolomide (NCT05128734), and topoisomerase inhibitors [96,97] have been proven effective in early clinical trials. Despite the moderate success, more effective therapies are still needed for breast cancers which are resistant to these RS inducers. In addition to the combination of different replication stress inducers, targeting the replication stress response signaling pathways is emerging as a promising strategy.

## 4. Targeting Replication Stress Response Signaling

If breast cancer cells with high levels of replicative lesions do not initiate cell death, they likely rely on RSR to provide sufficient time to deal with such lesions. The main RSR mediators for inducing cell-cycle delay or protecting stalled forks could, therefore, be promising targets (Table 1). Given the central role of ATR in preventing replication fork collapse, ATR inhibition initiates widespread DNA synthesis from dormant replication origins, generating ssDNA to exhaust the cellular pools of RPA [98,99]. ATR inhibitors (ATRi), such as AZD6738, BAY1895344, and M6620, have shown antitumor activity in preclinical and clinical studies [100]. Additional agent RP-3500 targeting ATR in breast cancer is currently under clinical phase 1 trial [72]. CHK1, the key downstream effector protein of the ATR response, plays an important role in triggering the S-phase checkpoint upon replication stress and preventing premature entry into mitosis. Currently, two CHK1 inhibitors, prexasertib [101] and GDC-0575 [74], have been reported to show preliminary antitumor activity in patients with breast cancer.

In addition to these two key players, the RSR also involves other important kinases, including WEE1, DNA-PK, and ATM, to arrest the cell cycle, protect stalled forks, and allow time for replication fork repair. For instance, the activation of ATR–CHK1 signaling can delay cell-cycle progression through WEE1 kinase [43]. A small-molecular inhibitor of WEE1 kinase, AZD1775, can increase unscheduled origin firings, leading to replication fork stalling and driving HER2-positive or TNBC breast cells into unscheduled mitosis [102,103]. Another WEE1 inhibitor ZN-c3 is undergoing evaluation in clinical trials for treatment of TNBC or metastatic breast cancer (NCT04158336, NCT05368506). The DNA-dependent protein kinase (DNA-PK), which has been well characterized in nonhomologous end-joining, has recently been shown to be required for efficient replication restart of stalled forks and ATR signaling [104,105]. Peposertib (M3814), an orally available DNA-PK inhibitor, is in clinical development and has shown modest efficacy in TNBC breast cancer [80]. AZD7648, a highly specific and potent DNA-PK inhibitor, is now in phase 1 clinical trial testing in patients with advanced cancers including breast cancer [81]. Additionally, ATM has been identified to play a replication stress-specific role in preserving replication fork integrity and maintaining DNA replication [106]. Currently, ATM inhibitors are under development and have been shown to be potent radiosensitizers in preclinical studies [82]. Among these ATM inhibitors, AZD0156 is currently being evaluated in phase 1 studies (NCT02588105). It is important to point out that the efficacy of these inhibitors as monotherapies depends on their ability to exploit the intrinsically high levels of replication stress within tumor cells and the ability to identify potential biomarkers to enhance sensitivity.

Limitations associated with the use of these inhibitors, such as excessive toxicity at effective doses and resistance to the treatment through compensatory pathways, promote the development of combination therapies. ATR and CHK1 inhibitors synergize with compounds that induce replication stress in breast cancers, including nucleoside analogues, platinum-based agents, and PARP inhibitors [107,108]. Additionally, a preclinical study showed that DNA-PK inhibitor AZD7648 enhances the efficiency of doxorubicin and PARP inhibitors in breast cancer cell lines and TNBC patient-derived xenograft models [109]. Clinical trials evaluating the antitumor efficacy of combining DNA-PK inhibitor and CHK1 inhibitor in breast cancer are ongoing (NCT04032080 and NCT02124148). Recently, a phase 2 clinical trial demonstrated that the WEE1 inhibitor adavosertib combined with cisplatin improved clinical outcomes for patients with metastatic TNBC [110]. Interestingly, dual inhibition of WEE1 and ATR sensitizes TNBC cells to cisplatin and PARP inhibitors [111], extending the catalog of possible combinations with other targeted inhibitors that could be implemented.

Despite these encouraging findings, challenges remain in determining the optimal therapeutic strategies, both monotherapy and combination therapy, to balance dose-dependent toxicity and antitumor efficiency. Another promising therapeutic strategy is the combination of agents targeting RS with immunotherapeutic agents, which share nonoverlapping toxicity profiles with agents targeting RS.

## 5. Emerging Combination Strategies of Immunotherapy with Agents Targeting RS

Immunotherapy, especially immune checkpoint blockade (ICB), is emerging as a new treatment modality in breast cancer. Monotherapy using antibodies against programmed death-1 (PD-1) and programmed death ligand-1 (PD-L1) reported objective response rates (ORRs) of around 10% to 20% in patients with metastatic breast cancer [112,113]. The lack of response of patients with breast cancer to immunotherapy has directed research toward novel combination therapeutic strategies aimed at transforming a higher proportion of non-responders into responders.

Preclinical studies have shown the link between immune response and pathways involved in RS. Accumulating evidence indicates that RS triggers the immune response through the accumulation of cytosolic DNA derived from the nucleus and the activation of DNA sensing pathways [21,114]. For instance, S-phase-specific DNA damage in breast cancer was identified to be associated with increased T-cell infiltration and PD-L1 expression in a STING-dependent manner [115]. Additionally, Diamond et al. showed that irradiated breast cancer cells transferred cytosolic dsDNA to dendritic cells and stimulated dendritic cell upregulation of costimulatory molecules and STING-dependent activation of IFN signaling [116]. Recently, a preclinical study reported that induction of RSR defects by CHK1/2 inhibition improved ICB response in murine breast cancer models [117]. Furthermore, other recent studies have illustrated how key RSR members including ATR, CHK1, and DNA-PK regulate immune response in breast cancer [118,119,120]. These studies have, thus, suggested that targeting RS would amplify the response of patients with breast cancer to ICB.

On the basis of these promising data in preclinical settings, combination strategies integrating drugs targeting RS with immunotherapies have advanced to clinical trials (Table 2). For example, the TOPACIO/KEYNOTE-162 trial reported that the anti-PD-1 plus PARP inhibitor combination therapy exhibited a good tolerability profile, with the ORR of 45% being higher in BRCA1/2 mutant TNBC patients than in nonmutant ones [121]. Meanwhile, the MEDIOLA trial showed that a combination of PARPi and anti-PD-L1 led to enhanced antitumor activity in one of the four cohorts for patients with advanced-stage BRCA1/2-mutant breast cancers [122]. Additionally, several ongoing trials are evaluating combinations of ATRi, DNA-PKi, or WEE1i with ICB therapy in breast cancer [123,124,125]. These studies would support the use of replication stress as a predictive marker for immunotherapy efficacy.

Despite these robust associations, a conflicting study reported that inhibition of RSR checkpoint kinases such as ATM, ATR, or CHK1 suppressed PD-L1 upregulation [126]. In addition, Burleign et al. identified DNA-PK as a DNA sensor that plays an essential role in the activation a STING-independent DNA sensing pathway [127]. Inhibition of DNA-PK led to the complete inhibition of this STING-independent DNA sensing pathway. In this context, blunting of this DNA sensing pathway by DNA-PK inhibition may be counterproductive to the activation of innate immunity. Together, these studies demonstrate that the mechanisms underlying the interplay between replication stress response and tumor immunogenicity have not yet been completely elucidated, and further research is required to provide insight into how these responses can be modulated optimally.

## 6. Predictive Biomarkers for Therapies Targeting Replication Stress

Optimal design of therapeutic strategies targeting RS requires reliable predictive biomarkers that can help to select, before the initiation of treatments, patients with breast cancer who would be most likely to benefit. Currently, there are a few biomarkers that have been identified to predict the response of patients with breast cancer to drugs enhancing RS. For example, Birkbak et al. reported that the expression levels of the BLM and FANCI genes are potential biomarkers that predict response of TNBC to platinum-based therapy [128]. Another recent report also suggested that the expression level of EZH2 can be used to identify patients with breast cancer who may benefit from platinum-based therapy [129]. They found that EZH2 inhibition enhanced the response of BRCA1-deficient breast tumors to platinum drugs. These two studies identified different biomarkers for platinum-based therapy in patients with breast cancer, suggesting that a single-gene function dependency usually represents a limited number of cases. Biomarkers identified in preclinical studies for predicting response of patients with breast cancer to RS-inducing agents need to be validated with clinical biopsy samples before being considered as useful biomarkers.

On the basis of positive outcomes in clinical trials, PARP inhibitors olaparib and talazoparib are approved as monotherapies for the treatment of patients with germline BRCA-mutated, HER2-negative advanced or metastatic breast cancer [130,131,132]. However, PARPi resistance has proved to be a major problem in the clinic [133]. The mechanisms underlying this resistance have been characterized, and restoration of replication fork stability is one of major mechanisms for PARPi resistance [133]. Collectively, these studies suggest that, owing to the complexity of tumor microenvironment, single biomarkers are insufficient to accurately predict clinical outcomes with therapies targeting RS, and a combination of genetic deficiencies will be required to deliver a sufficient degree of sensitivity to drugs targeting RS.

In addition to these biomarkers for predicting response to RS-inducing agents, other genomic aberrations have been identified as potential markers for evaluating the response of breast cancer patients to drugs targeting RSR signaling. In a preclinical study, ARID1A deficiency was found to sensitize breast cancer cells to ATR inhibitor both in vivo and in vitro by causing topoisomerase 2A and cell-cycle defects [134]. This study indicates that ARID1 defects may serve as a biomarker of single-agent ATR inhibitor response. Additionally, inhibition of RAD51 has been demonstrated to increase dependency on ATR-CHK1-mediated RSR signaling, and subsequent inhibition of ATR or CHK1 results in preferentially killing of HR-deficient breast cancer cells [135]. It is important to point out that these biomarkers identified in preclinical models need to be validated using clinical samples. A functional genetic screen showed that silencing of Fanconi anemia and HR genes resulted in increased replication stress and nucleotide depletion after WEE1 inhibition, culminating in unscheduled mitotic entry [136]. On the basis of this preclinical model, a clinical trial is ongoing using alterations in homologous recombination repair-related genes as selective biomarkers for patients with advanced breast cancer (NCT03330847) [137]. Currently, there are few biomarkers that effectively predict the response of patients with breast cancer to RSR inhibitors.

Unlike studies using a sole predictive biomarker, McGrail et al. developed a gene signature which can predicts RSR defects (RSRDs) in patient breast hyperplasias and cell lines [5]. Importantly, they found the RSRD gene signature can accurately identify patients with non-hypermutated cancer across seven tumor types, including breast cancer, who may benefit from ICB [117]. Although this study has not yet been validated using clinical samples, such an RSRD signature may provide broader means of patient selection and could become relevant as potential biomarkers if validated in the clinic.

One key question remains to be addressed, when identifying biomarkers that indicate a dependency on the RSR and a likely response to inhibitors targeting key RSR kinases, is whether the clinical biopsy taken at initial diagnosis still indicates the level of RSR dependence in the tumor to be treated. Therefore, genetic analysis of clinical samples obtained at different timepoints, i.e., pre- and post-treatment tumor samples, may represent an important approach for understanding the dynamic changes of response to agents targeting RSR, and an additional goal would be the identification of more reliable predictive biomarkers that can help to select patients prior to therapy.

## 7. Conclusions and Future Perspectives

In this review, we highlighted the advances in understanding the mechanism of the cellular response to replication stress and development of promising drug candidates targeting RS. Despite these significant advancements, challenges remain in discerning the optimal therapeutic strategies. For instance, the applicability and efficacy of these drugs that enhance endogenous RS or target RSR is limited by their associated toxicities and the high rate of drug resistance [20,21,138]. As noted, these therapies can induce apoptosis in normal cells within the gut epithelium and bone marrow, leading to undesirable and intolerable side-effects [139,140].

In fact, clinical studies assessing the potential of drugs targeting RSR pathways in breast cancer, both as monotherapy and as combination therapy described above, are still in the very early stages; the antitumor activity of these drugs has not yet been completely elucidated. Additionally, whether the combination of these drugs with immunotherapeutic agents is as effective and less toxic than the combination with DDR agents needs to be addressed.

Another important limitation is the current technologies available to personalize and define patient-specific RS in real time in the clinic. Potential predictive biomarkers have been identified to select patients who are most likely to benefit from certain therapies. However, only a few biomarkers are currently FDA-approved for clinic use. Other emerging biomarker candidates have shown their unique advantages, as well as limitations, in both preclinical and clinical studies [7,43]. Indeed, novel technologies are needed to assist precision medicine approaches. Genome-wide screening using CRISPR/Cas9 technology performed in more physiologically relevant settings, such as in 3D organoid cultures or in vivo mouse models, will likely identify novel clinically relevant targets in DNA replication and RSR.

Targeting replication stress has shown promise in breast cancer treatment. Future research in this area would likely provide insightful clues for identification of novel molecular targets in DNA replication and RSR, for finding the optimal multimodality therapy, as well as for discovering additional biomarkers that can collectively predict RS phenotype and maximize clinical benefit.

## Figures and Tables

**Table 1:** Therapeutic agents that enhance replication stress in breast cancer.

Class of Agents or Target	Mechanism of Action	Compounds	Clinical Stage	Limitations	Refs.
Nucleoside analogues	Incorporation into DNA to inhibit replication	5-Fluorouracil	Approved		[58]
		Gemcitabine	Approved		[21]
		Azacitidine	Phase 2		[59]
Alkylating agents and platinum compounds	Modification of DNA	Carboplatin	Phase 2 and 3		[60]
		Cisplatin	Phase 2		[61]
		Cyclophosphamide	Approved		[21]
		Methotrexate	Approved		[21]
		Temozolomide	Phase 2	Lack of systemic activity for temozolomide in a heavily pretreated, heterogeneous group of breast cancer patients	[62,63]
Topoisomerase I and II	Relaxation of DNA supercoiling	Doxorubicin	Approved		[21]
		Epirubicin	Approved		
		Etoposide	Phase 2	Dose-limiting toxicities	[60]
		Mitoxantrone	Phase 2		[21]
PARP	Recruitment of MRE11 to stalled reverse forks, regulation of fork restart, ssDNA break repair, and regulation of fork elongation	Olaparib	Approved		[64,65,66]
		Niraparib	Approved		
		Rucaparib	Approved		
		Talazoparib	Approved		
		Veliparib	Phase 3	Veliparib plus carboplatin-containing chemotherapy did not impact long-term outcomes in TNBC	
		RP12146	Phase 1/1b		
DNA polymerases	Regulation of DNA replication	CD437	Preclinical		[67]
		Zelpolib	Preclinical		[68]
DNA2	Regulation of Fork restart and ART activation	C5	Preclinical		[69]
		NSC-105808	Preclinical		[37]
RAD52	Regulator of initiation and resolution of fork reversal	D-G23	Preclinical		[70,71]
		D-I03	Preclinical		
		F79	Preclinical		
		A5MP	Preclinical		
		AICAR	Preclinical		
ATR	Essential kinase in replication stress response	M4344	Phase 1		[72,73]
		BAY1895344	Phase 1	Dose-limiting hematological toxicities	
		RP-3500	Phase 1		
		AZD6738	Phase 1 and 2	Toxicities such as hematological toxicities and immune toxicities were evident in ≥20% of subjects	
		M6620	Phase 1 and 2		
CHK1	Key downstream effector kinase of the ATR response	GDC-0575	Phase 1		[74]
		Prexasertib	Phase 2		[7]
		UCN-01	Phase 2	The clinical outcome was inconclusive	[75]
		AZD7762	Phase 1	The development was stopped due to cardiac toxicity	[76]
WEE1	G2/M checkpoint kinase	AZD1775	Phase 2		[77]
		ZN-c3	Phase 1		[78]
		Debio0123	Phase 1		[79]
DNA-PK	Regulation of stalled forks restart and ATR signaling	M3814	Phase 1		[80]
		AZD7648	Phase 1		[81]
ATM	Preserving replication fork integrity and maintaining DNA replication	AZD0156	Phase 1	Dose-limiting hematological toxicities	[82]

**Table 2:** Combination strategies of ICB with agents targeting RS in breast cancer.

Class of Agents or Target	Compounds	Trials	Clinical Stage	Biomarker
Alkylating agents and platinum compounds	Carboplatin + pembrolizumab	NCT03121352	Phase 2	N/A
		NCT03639948	Phase 2	N/A
	Cyclophosphamide + pembrolizumab	NCT03139851	Phase 2	N/A
		NCT02768701	Phase 2	N/A
		NCT03971045	Phase 2	N/A
PARP	Niraparib + pembrolizumab	NCT02657889	Phase ½	BRCA1/2 mutant
	Niraparib + dostarlimab	NCT04673448	Phase 1	BRCA mutant
		NCT04837209	Phase 2	N/A
	Olaparib + pembrolizumab	NCT03025035	Phase 2	BRCA mutant or HDR-defect
	Olaparib + atezolizumab	NCT02849496	Phase 2	BRCA mutant HER2-negative
	Talazoparib + avelumb	NCT03330405	Phase ½	N/A
		NCT03964532	Phase ½	N/A
	Olaparib + durvalumab	NCT03544125	Phase 1	N/A
		NCT02484404	Phase ½	N/A
		NCT03801369	Phase 2	N/A
		NCT05498155	Phase 2	BRCA mutant HER2-negative
		NCT03167619	Phase 2	N/A
ATR	AZD6738 + durvalumab	NCT03740893	Phase 2	N/A
WEE1	ZN-c3 + pembrolizumab	NCT05431582	Phase 1	N/A
DNA-PK	M3814 + avelumab	NCT03724890	Phase 1	N/A

## Data Availability

Not applicable.
